# Supplementary material for: Whole-Genome Amplification—Surveying Yield, Reproducibility, and Heterozygous Balance, Reported by STR-Targeting MIPs
Source: Int J Mol Sci. 2022 May 31;23(11):6161. doi: 10.3390/ijms23116161 (PMC9181316; doi:10.3390/ijms23116161)
Supplement: Supplementary file 1 [file ijms-23-06161-s001.zip › ijms-1728313-supplementary.pdf]

Supplementary Figures

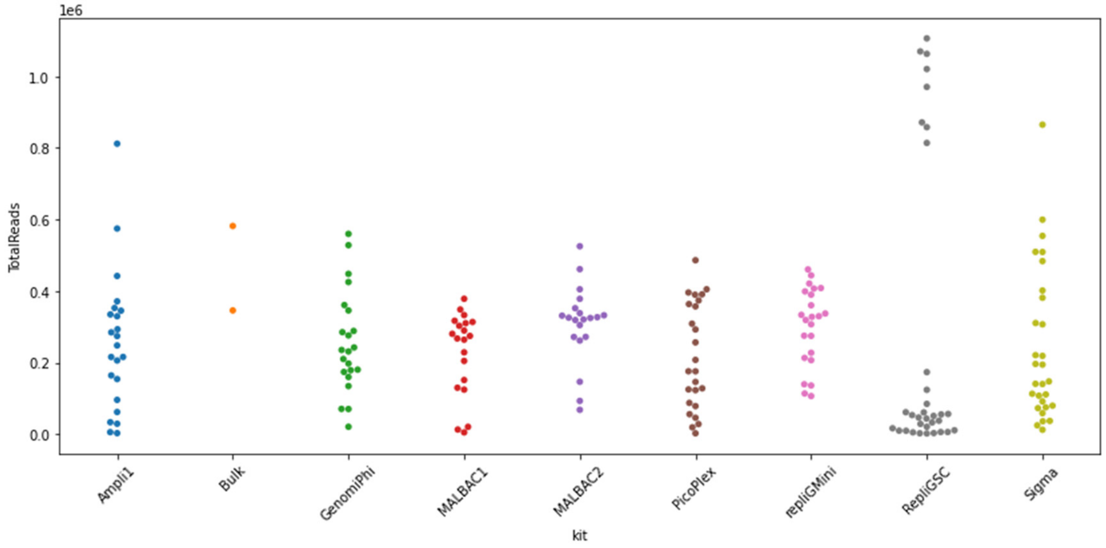

Figure S1. Total coverage—the number of NGS reads per cell barcode.

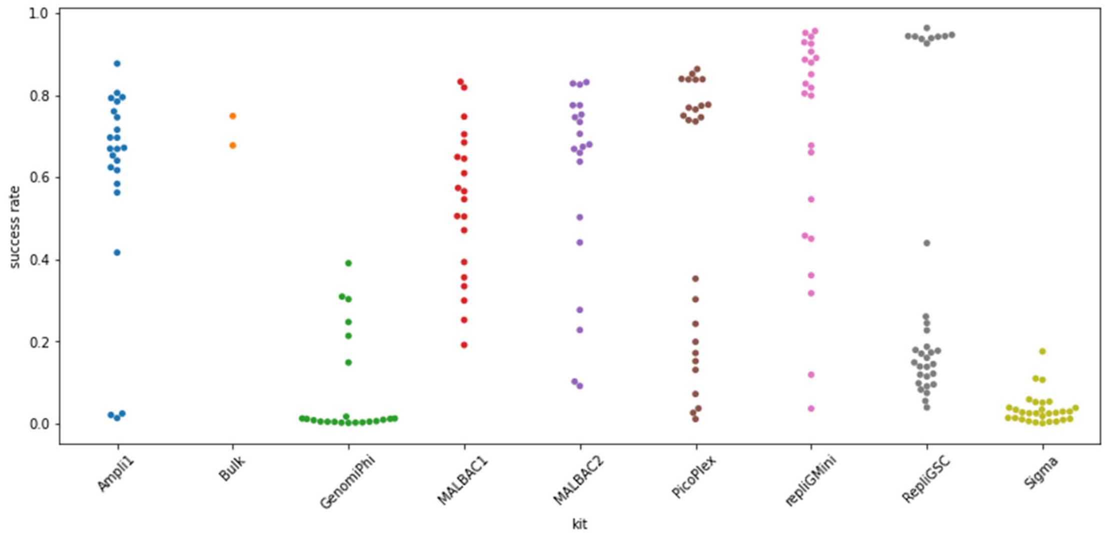

Figure S2. Mapping rates against a custom reference genome for the STR-targeting panel used here. Success rate (Y-axis) indicates the fraction of total reads (Supplementary Figure S1) that mapped to the index.

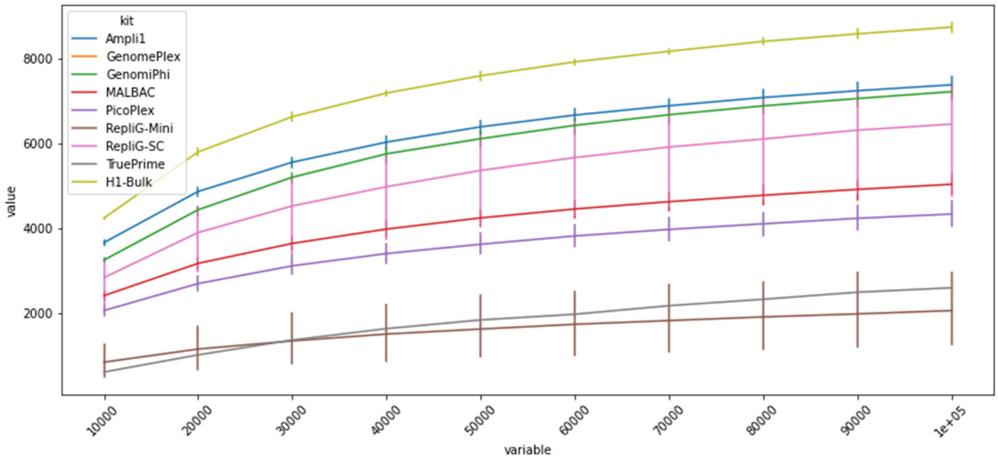

Figure S3. Loci sampled per cell (higher is better)—amplicon coverage per single cell per kit (Y-axis), per simulated coverage (in #reads, X-axis). As NGS coverage may be considered an orthogonal experimental parameter that may be controlled for by sample dilution and normalized from the results, fixed numbers of NGS reads are sampled from the mapped reads population. This corrects for library concentration biases but also compensates for samples that mapped poorly.

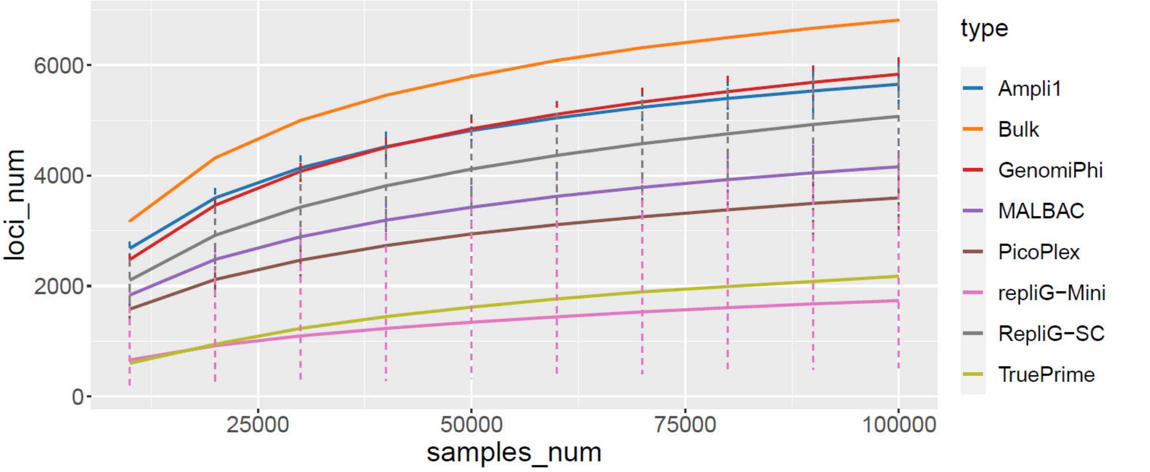

Figure S4. Loci sampled per cell (higher is better)—amplicon coverage per single cell per kit (Y-axis), per simulated coverage (in #reads, X-axis). Similar to Supplementary Figure S3, but with additional normalization for mapping rate.

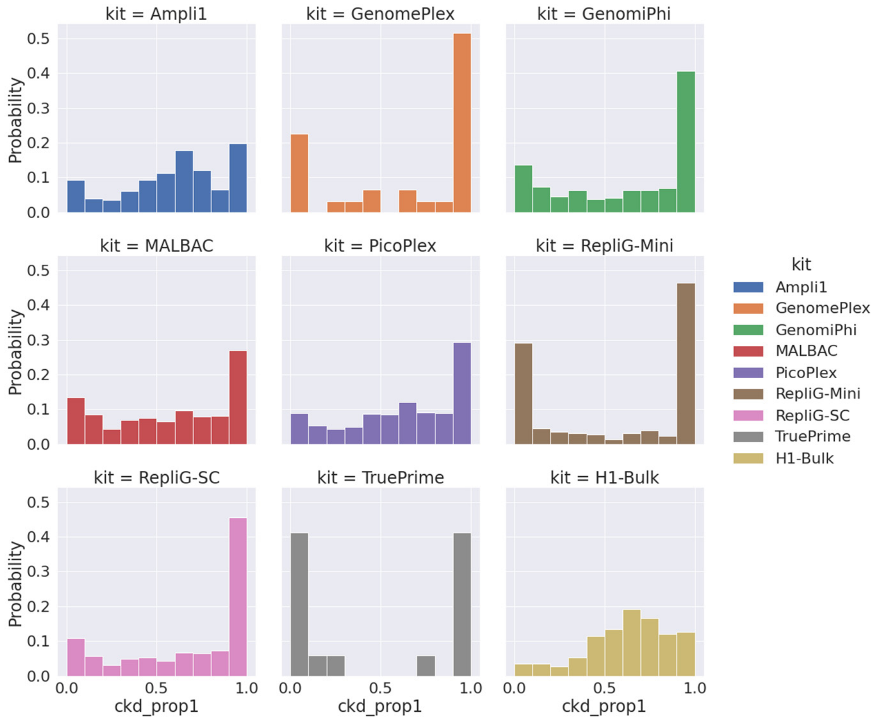

Figure S5. Allelic balance (closer to 0.5 is better)—alternative view of Figure 3 with distribution of proportion between alleles at heterozygous loci, where 0.5 indicates a perfectly balanced result, while 0/1 indicates worst case.
